# Platelet factors are induced by longevity factor klotho and enhance cognition in young and aging mice

**DOI:** 10.1038/s43587-023-00468-0

**Published:** 2023-08-16

**Authors:** Cana Park, Oliver Hahn, Shweta Gupta, Arturo J. Moreno, Francesca Marino, Blen Kedir, Dan Wang, Saul A. Villeda, Tony Wyss-Coray, Dena B. Dubal

**Affiliations:** 1 Department of Neurology and Weill Institute for Neurosciences, University of California, San Francisco, CA USA; 2 Department of Neurology and Neurological Sciences, Stanford University School of Medicine, Stanford, CA USA; 3 Neurosciences Graduate Program, University of California, San Francisco, CA USA; 4 Department of Anatomy, University of California, San Francisco, CA USA; 5 Department of Physical Therapy and Rehabilitation Science, San Francisco, CA USA; 6 Eli and Edythe Broad Center for Regeneration Medicine and Stem Cell Research, San Francisco, CA USA; 7 Biomedical Sciences Graduate Program, University of California, San Francisco, CA USA; 8 The Knight Initiative for Brain Resilience, Stanford University, Stanford, CA USA; 9 Paul F. Glenn Center for the Biology of Aging, Stanford University School of Medicine, Stanford, CA USA; 10 Wu Tsai Neurosciences Institute, Stanford University, Stanford, CA USA

**Keywords:** Cognitive ageing, Spatial memory, Learning and memory

## Abstract

Platelet factors regulate wound healing and can signal from the blood to the brain^1,2^. However, whether platelet factors modulate cognition, a highly valued and central manifestation of brain function, is unknown. Here we show that systemic platelet factor 4 (PF4) permeates the brain and enhances cognition. We found that, in mice, peripheral administration of klotho, a longevity and cognition-enhancing protein^3–7^, increased the levels of multiple platelet factors in plasma, including PF4. A pharmacologic intervention that inhibits platelet activation blocked klotho-mediated cognitive enhancement, indicating that klotho may require platelets to enhance cognition. To directly test the effects of platelet factors on the brain, we treated mice with vehicle or systemic PF4. In young mice, PF4 enhanced synaptic plasticity and cognition. In old mice, PF4 decreased cognitive deficits and restored aging-induced increases of select factors associated with cognitive performance in the hippocampus. The effects of klotho on cognition were still present in mice lacking PF4, suggesting this platelet factor is sufficient to enhance cognition but not necessary for the effects of klotho—and that other unidentified factors probably contribute. Augmenting platelet factors, possible messengers of klotho, may enhance cognition in the young brain and decrease cognitive deficits in the aging brain.

## Abstract (web summary)

The authors found that klotho, a longevity and cognition-enhancing factor, can increase the levels of multiple platelet factors in plasma, including PF4. PF4 treatment, which permeated the brain, in turn, enhanced cognition in young and old mice.

## Main

Platelets are small, anuclear blood cells that store bioactive factors in specialized cytoplasmic compartments^2^. Upon environmental stimulation such as exercise, tissue injury or stress, varying forms of platelet activation cause context-dependent and selective release of contents. Thus, diverse forms of platelet activation transduce fundamental biologic actions ranging from hemostasis to neurogenesis^1^. Likewise, platelet dysfunction is implicated in inflammation, bleeding and central nervous system diseases^8^. The idea that platelets could be messengers of brain health is supported by observations that exercise activates platelets and subsequent release of platelet factor 4 (PF4) supports hippocampal neurogenesis^1^. However, whether platelet factors—defined as proteins released from platelet granules and lysosomes upon platelet activation—could modulate cognition itself, a highly valued and central manifestation of brain function that declines with aging and disease, is unknown. This is an important knowledge gap since cognitive dysfunction is among our biggest biomedical challenges with no effective treatments. We thus investigated platelet factor function on underlying substrates of cognition, and on cognition itself.

Klotho is a longevity factor^9–11^ that improves cognitive functions^3–5,7,12,13^. It circulates as a hormone following cleavage from its transmembrane form and impacts insulin^14^, fibroblastic growth factor (FGF)^15^, Wnt^16^ and *N*-methyl-d-aspartate receptor (NMDAR) signaling^3–5^. Human genetic variation of *KLOTHO* increases its systemic levels^3,17,18^ and associates with enhanced brain connectivity^17^ and cognition^3,17,19,20^ in aging human populations. In experimental studies of mice, acute and systemic elevation of the secreted form of α-klotho (klotho) increases synaptic plasticity, cognition and neural resilience^5,21^. Highlighting its therapeutic potential, systemic treatment with klotho levels present during the human lifespan also enhances cognition in the aging nonhuman primate brain^6^—in the context of increased genetic, anatomic and functional complexity. Since peripherally injected klotho does not cross into the brain^5,22^, peripheral messengers that transduce its signals remain to be identified. We therefore investigated potential klotho-related cognitive signals—and unexpectedly encountered platelet factors.

## Longevity factor klotho increases systemic levels of platelet factors

**Fig. 1:**
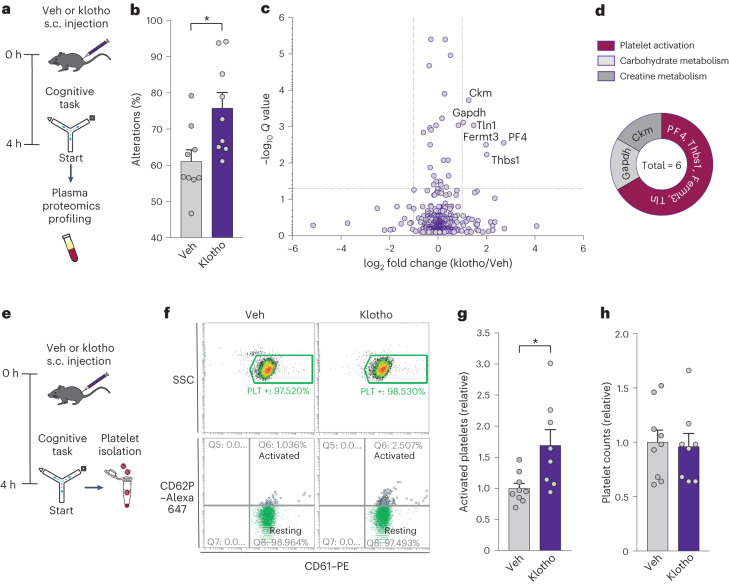
Klotho induces platelet activation in the blood and increases circulating platelet factors. **a**, Paradigm for plasma proteomics profiling. Young mice (male, age 4 months) were treated with either vehicle (Veh) (*n* = 10 mice) or klotho (*n* = 9 mice) (s.c., 10 μg kg^−1^) followed by plasma proteomics analysis. **b**, Percentage of alternations among arms during exploration of the Y maze. Young mice (male, age 4 months, *n* = 9 mice per group) were treated with either Veh or klotho. *P = 0.014 (two-tailed *t*-test). **c**, Plasma proteomics by mass spectrometry analysis 4 h after treatment with Veh or klotho identified six differentially expressed proteins (*Q* < 0.05, dashed horizontal line; and fold change >2, dashed vertical line). **d**, Canonical functions of top six differentially expressed plasma proteins following klotho treatment (*Q* < 0.05 and fold change >2). **e**, Paradigm for measuring platelet activation. Young mice (male, age 5 months, *n* = 8–9 mice per group) were treated with either Veh or klotho (s.c., 10 μg kg^−1^) followed by platelet isolation from whole blood and then platelet activation analysis by FACS with markers CD61 and CD62P. **f**, Flow cytometry plots from FACS showing platelet populations. The upper graphs show density plots of the platelets, gated by side scatter (SSC) (for granularity) and CD61 positivity. The lower graphs show dot plots of the percentage activated (CD61 and CD62P-positive) and resting (CD61-positive only) platelets. **g**, Quantification of activated platelets in young mice following treatment with Veh (*n* = 9 mice) or klotho (*n* = 8 mice). *P = 0.014 (two-tailed *t*-test). **h**, Quantification of platelets counts in young mice following treatment with Veh (*n* = 9 mice) or klotho (*n* = 8 mice). Data are presented as mean ± s.e.m. Source data

An untargeted proteomic analysis identified platelet biology as a target of klotho. We hypothesized that klotho engages peripheral messengers that transduce signals into the brain. To identify klotho-related cognitive signals, we performed an untargeted mass spectrometry-based proteomic profiling of plasma isolated from mice 4 h following peripheral treatment with vehicle or klotho and a cognitive task, exploration of a Y maze (Fig. 1a). As expected^5,6,21^, klotho enhanced cognition (Fig. 1b) within 4 h, when systemic klotho levels were increased by approximately five-fold^6^. Quantitative analysis of plasma proteomics identified that klotho increased several factors (Fig. 1c and Supplementary Table 1); of the top six (*Q* value, *P* < 0.05, >2 fold increase), 66% were platelet factors resulting from platelet activation and subsequent α-granule release (Fig. 1d). These findings suggested that systemic klotho influences platelets; specifically, it suggested that klotho could activate platelets and increase expression of proteins released from platelets.

**Extended Data Fig. 1:**
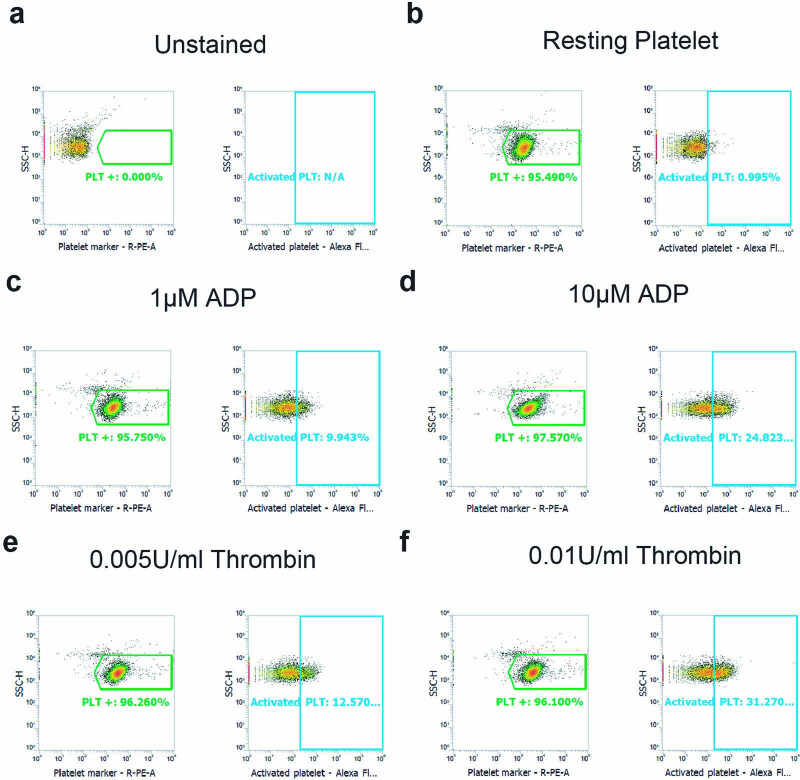
Representative FACS gating strategy for identifying platelet populations from a young mouse (linked to Fig. 1f). (**a**) Isolated platelets unstimulated and stained CD61 (platelet marker) and CD61P (platelet activation marker). (**b**) Resting platelet population (PLT + , green) shows a shift of population to the CD61-positive (PLT + , green) compared to unstained platelets (**a**). The activated platelet population (Activated PLT, cyan) is set to around 1% by adjusting CD62P fluorescence intensity. Isolated platelets stained with CD61 and CD62P but not stimulated with any platelet agonists. (**c**,**d**) ADP treatment or (**e,f**) Thrombin treatment results in a shift of the platelet-positive population (PLT + , green) to the CD62P-positive (platelet activation positive, Activated PLT, cyan) platelets in a dose-dependent manner. Isolated platelets were stimulated with two different doses of platelet agonists, ADP or Thrombin, prior to co-labeling with CD61 and CD62P.

We next tested whether systemic klotho treatment can activate platelets. Mice underwent vehicle or klotho treatment; 4 h later, following exploration of a Y maze, platelets were immediately isolated from whole blood and sorted by fluorescence-activated cell sorting (FACS) (Fig. 1e). We quantified platelet activation levels by flow cytometry^8,23^, expressed as the percentage of activated platelets (CD62P positive) within the total platelet population (CD61 positive), using established methods^1,24,25^. Resting levels of platelet activation were derived from platelets isolated from mice without addition of any platelet-activating agonists. Acute klotho treatment followed by a cognitive task nearly doubled the resting level of platelet activation (Fig. 1f,g and Extended Data Fig. 1) without altering the number of platelets (Fig. 1h). Levels of activation were lower than those following known agonist exposure^26,27^ or thrombosis^28,29^. It should be noted that activated platelets marked by CD62P (or P-selectin) are rapidly cleared from peripheral blood. Thus, these results may underestimate the amount of activated platelets or, alternatively, include some platelet activation during sample preparation. Despite these caveats, systemic klotho treatment significantly increased platelet activation.

**Extended Data Fig. 2:**
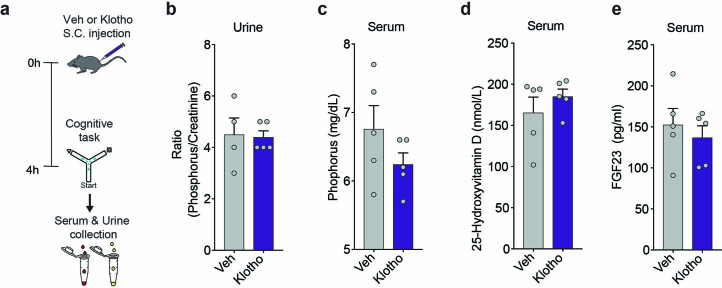
Acute klotho treatment does not alter the FGF23-klotho endocrine system, renal phosphate, or vitamin D (linked to Fig. 1f,g). (**a**) Paradigm for serum and urine profiling. Young mice (male, age 4 months; n = 4-5 mice per group) were treated with either Veh or Klotho (s.c. 10 μg/kg) followed by serum and urine collection and profiling. (**b**) Urine ratio of phosphorus to creatinine in urine between groups (n = 4 mice for Veh group; n = 5 mice for Klotho group). (**c**) Serum Phosphorus levels between groups (n = 5 mice per group). (**d**) Serum 25-Hydroxyvitamin D levels between groups (n = 5 mice per group). (**e**) Serum FGF23 levels between groups (n = 5 mice per group). Data are presented as means ± SEM. Source data

**Extended Data Fig. 3:**
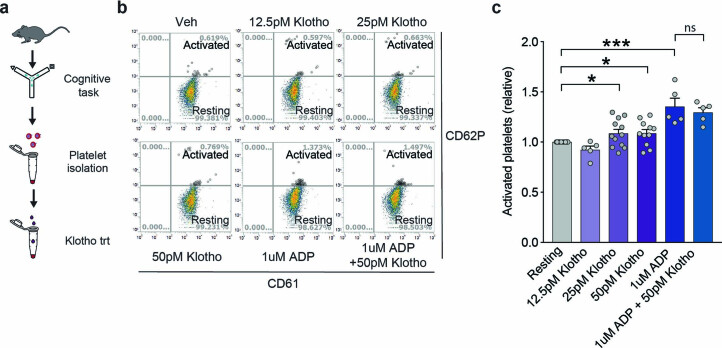
Klotho modestly but directly activates platelets in a potentially ADP-dependent pathway (linked to Fig. 1f,g). (**a**) Experimental paradigm of measurement of platelet activation with *in vitro* klotho treatment. Platelets were isolated from young mice (male, age 6 months, n = 5-12 mice per group) and were treated with klotho. Platelet activation levels were measured by FACS with markers CD61 and CD62P. (**b**) Representative flow cytometry plots from FACS showing platelet populations in the experimental groups. Graphs show dot plots of the percentage activated (CD61 and CD62P-positive) and resting (CD61-positive only) platelets. (**c**) Quantification of activated platelets following *in vitro* klotho treatment of isolated platelets (n = 12 mice for Resting; n = 5 mice for 12.5pM Klotho; n = 12 mice for 25pM Klotho; n = 12 mice for 50pM Klotho; n = 5 mice for 1uM ADP; n = 5 mice for 1uM ADP +50pM Klotho). *P = 0.041 (Resting vs 25pM of Klotho), *P = 0.021 (Resting vs 50pM of Klotho), ***P < 0.001 (Resting vs ADP) (two-tailed t-tests)(Benjamini & Hochberg). Data are presented as means ± SEM. Source data

We wondered how klotho activates platelets. We first measured if it might acutely increase plasma FGF23, as observed in long-term transgenic klotho overexpression^30^. Four hours following acute vehicle or klotho treatment—at the same time of klotho-mediated platelet activation and cognitive enhancement—there were no differences in systemic levels of FGF23, vitamin D, phosphorous or other related measures between the groups (Extended Data Fig. 2). This suggests that klotho probably activates platelets through FGF23-independent pathways. We then investigated whether klotho can directly activate isolated platelets, in the absence of other plasma factors. We collected platelets from mice following a cognitive task and added vehicle or klotho onto the isolated platelets in vitro (Extended Data Fig. 3a,b) followed by FACS analysis. Direct application of klotho weakly activated isolated platelets (Extended Data Fig. 3c), and did so in a manner potentially dependent on ADP (Extended Data Fig. 3c). Notably, these in vitro data do not rule out other indirect mechanisms linking klotho to platelet activation. It is interesting to speculate that weak agonism of platelets, such as with klotho, may serve to modestly activate release of alpha granules, and thus release chemokines—but ultimately avoid hemostasis and clot formation. How low levels of klotho-mediated platelet activation could differ or converge upon other paths to platelet activation, such as through exercise, remains to be investigated.

## Klotho-mediated cognitive enhancement may require platelet activation

To follow on the finding that klotho activates platelets, we tested if platelet activation may be necessary for klotho-mediated cognitive enhancement. We implemented pharmacologic platelet inhibition, requiring at least 3 days of oral aspirin (ASA) and clopidogrel (CPG) administration^31,32^. It should be noted that ASA/CPG is a robust, but not specific, inhibitor of platelet activation used for human cerebrovascular and cardiovascular therapies. While CPG more precisely targets the P2RY12 purinergic receptor on platelets to block ADP-mediated platelet activation^33^, ASA inhibits COX1 largely in platelets but also throughout the body, among its many actions^34^. Despite this caveat, this treatment paradigm is a powerful blocker of platelet activation, even in mice^31,32^.

**Fig. 2:**
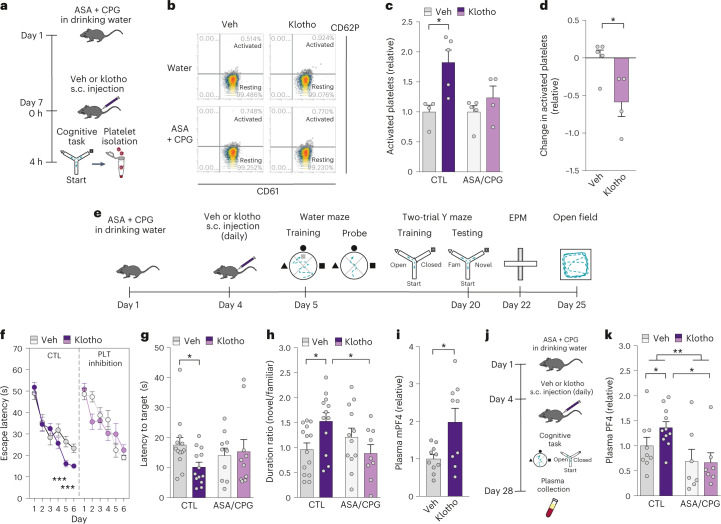
ASA/CPG treatment inhibits platelet activation and blocks klotho-mediated cognitive enhancement. **a**, Paradigm of treatment by aspirin (ASA, 0.4 mg ml^−1^ in drinking water) and clopidogrel (CPG, 0.15 mg ml^−1^ in drinking water) followed by treatment with vehicle (Veh) or klotho (s.c., 10 μg kg^−1^) in young mice (male, age 6 months, *n* = 4–5 mice per group). **b**, Flow cytometry plots from FACS showing platelet (PLT) populations in the experimental groups. Graphs show dot plots of the percentage activated (CD61 and CD62P-positive) and resting (CD61-positive only) platelets. **c**, Quantification of activated platelets in young mice with (ASA/CPG) or without (control, CTL) platelet inhibition following Veh or klotho treatment (*n* = 4 mice for CTL/Veh; *n* = 5 mice for CTL/klotho; *n* = 5 mice for ASA/CPG/Veh; *n* = 4 mice for ASA/CGP/klotho). Two-way ANOVA: interaction *P* = 0.092. *P = 0.028 (two-tailed *t*-test) (Benjamini–Hochberg). **d**, Change in activated platelets with klotho treatment (*n* = 5 mice for Veh; *n* = 4 mice for klotho). *P = 0.025 (two-tailed *t*-test). **e**, Paradigm of ASA/CPG administration and Veh or klotho treatment (s.c. 10 μg kg^−1^, daily) followed by cognitive testing (male, age 4 months, *n* = 9–14 mice per group) (elevated plus maze, EPM). **f**, Spatial learning curves (platform hidden) of mice treated with Veh or klotho, with or without ASA/CPG, in the Morris water maze (*n* = 14 mice for CTL/Veh; *n* = 14 mice for CTL/klotho; *n* = 13 mice for ASA/CPG/Veh: *n* = 12 mice for ASA/CPG/klotho). Two-way ANOVA: interaction klotho by time ; (CTL) *P* = 0.006; ***P < 0.001 (two-tailed, paired *t*-test: days 5, 6); two-way ANOVA: klotho (CTL versus ASA/CPG) *P* = 0.029. **g**, Probe trial conducted 1 h after hidden platform training and removal of the escape platform (*n* = 13 mice for CTL/Veh; *n* = 13 mice for CTL/klotho; *n* = 10 mice for ASA/CPG/Veh; *n* = 10 mice for ASA/CPG/klotho). The latency to the target indicates memory. Two-way ANOVA: interaction *P* = 0.057. *P = 0.022 (two-tailed *t*-test). **h**, Spatial and working memory of young mice treated with Veh or klotho, with or without ASA/CPG, was assessed by the two-trail Y maze (*n* = 14 mice for CTL/Veh; *n* = 12 mice for CTL/klotho; *n* = 12 mice for ASA/CPG/Veh; and *n* = 9 mice for ASA/CPG/klotho). The ratio of duration spent in novel and familiar arms during testing was measured 16 h after training. Two-way ANOVA: interaction *P* = 0.010; *P = 0.026 (Veh versus klotho; CTL) (two-tailed *t*-test) (Benjamini–Hochberg); and *P = 0.018 (CTL versus ASA/CPG; klotho) (two-tailed *t*-test) (Benjamini–Hochberg). **i**, Quantification of mouse PF4 level by ELISA of plasma from young mice 4 h (male, age 4 months) following treatment with Veh (*n* = 10 mice) or klotho (*n* = 9 mice) (10 μg kg^−1^, s.c). *P = 0.016 (two-tailed *t*-test). **j**, Paradigm of plasma collection for PF4 ELISA from young mice (male, age 4 months, *n* = 7–11 mice per group) with either drinking water or ASA/CPG (in drinking water) and treatment with Veh or klotho (s.c., 10 μg kg^−1^), followed by testing in the Morris water maze, two-trial Y maze, elevated plus maze and open field. **k**, Quantification of mouse PF4 level by ELISA of plasma from young mice following platelet inhibition with or without treatment of Veh or klotho daily (s.c. 10 μg kg^−1^) (*n* = 9 mice for CTL/Veh; *n* = 11 mice for CTL/klotho; *n* = 7 mice for ASA/CPG/Veh; *n* = 7 mice for ASA/CPG/klotho). Two-way ANOVA: platelet inhibition **P = 0.007; *P = 0.042 (Veh versus klotho; CTL) (one-tailed *t*-test since replication) (Benjamini–Hochberg); *P = 0.017 (CTL versus ASA/CPG; klotho) (two-tailed *t*-test)(Benjamini–Hochberg). Data are presented as mean ± s.e.m. Source data

**Extended Data Fig. 4:**
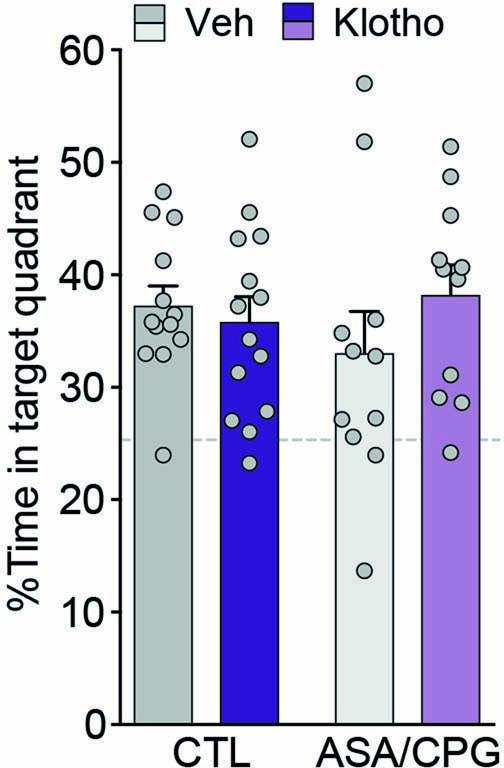
ASA/CPG administration with or without Klotho treatment does not alter time spent in target quadrant (linked to Fig. 2f). Probe trial conducted 1 hr after hidden platform training and removal of the escape platform (n = 13 mice for CTL/Veh; n = 14 mice for CTL/Klotho; n = 11 mice for ASA/CPG/Veh; n = 11 mice for ASA/CPG/Klotho). Percentage of time mice spent in the target quadrant of the maze is shown to indicate the memory of the platform location. The dashed line represents chance performance at 25%. Data are presented as means ± SEM. Source data

**Extended Data Fig. 5:**
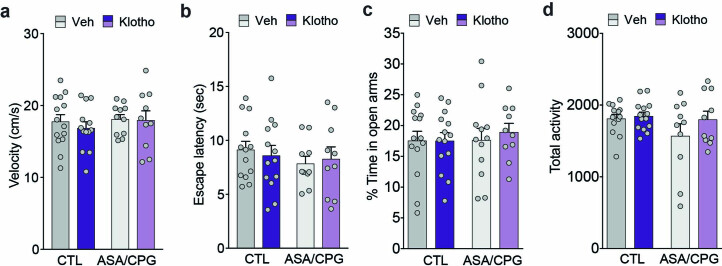
ASA/CPG administration with or without Klotho treatment does not alter swim speed, ability to find the target platform, anxiety-like behavior, or total activity (linked to Fig. 2e–h). (**a**) Swim speed of mice was measured in the water maze. Mice were treated with Veh or Klotho, with or without platelet inhibition (n = 14 mice for CTL/Veh; n = 13 mice for CTL/Klotho; n = 11 mice for ASA/CPG/Veh; n = 10 mice for ASA/CPG/Klotho). (**b**) Ability to find the target platform, identified by a visual cue was measured by the time spent in the water maze and averaged over two trials. Mice were treated with Veh or Klotho, with or without platelet inhibition (n = 13 mice for CTL/Veh; n = 13 mice for CTL/Klotho; n = 10 mice for ASA/CPG/Veh; n = 10 mice for ASA/CPG/Klotho). (**c**) Anxiety-like behavior of mice was measured by the percentage of time spent in the open arms of the elevated plus maze during a 10 min exploration period of mice (n = 10-14 mice per group) treated with Veh or Klotho, with or without platelet inhibition (n = 14 mice for CTL/Veh; n = 14 mice for CTL/Klotho; n = 12 mice for ASA/CPG/Veh; n = 10 mice for ASA/CPG/Klotho). (**d**) Hyperactivity was measured by the total movements during exploration of the open field for 10 min of mice treated with Veh or Klotho, with or without platelet inhibition (n = 13 mice for CTL/Veh; n = 14 mice for CTL/Klotho; n = 10 mice for ASA/CPG/Veh; and n = 10 mice for ASA/CPG/Klotho). Data are presented as means ± SEM. Source data

We next verified that pharmacologic platelet inhibition blocked klotho-mediated platelet activation (Fig. 2a–d). Using this paradigm, we then tested cognition in the Morris water maze and the two-trial Y maze (Fig. 2e). In the Morris water maze, as expected^5,21^, klotho treatment enhanced spatial learning and memory (Fig. 2f,g); remarkably, ASA/CPG completely blocked klotho-mediated learning (Fig. 2f). There were, however, no differences in time spent in target quadrants (Extended Data Fig. 4). There were also no differences in swim speed, or ability to find the target platform, identified by a visual cue (Extended Data Fig. 5a,b) among the groups—indicating a specific effect on cognition. In the two-trial Y maze, ASA/CPG blocked klotho-mediated cognitive enhancement by measures of learning and memory (Fig. 2h). Neither ASA/CPG nor klotho treatment altered measures of anxiety-like behavior in the elevated plus maze (Extended Data Fig. 5c) or of total activity in the open field (Extended Data Fig. 5d). Thus, this paradigm of pharmacologic platelet inhibition specifically blocked klotho-mediated cognitive enhancement measured by two cognitive tests without altering other behaviors. While a main target of ASA/CPG is blocking platelet activation, the contribution of nonspecific effects of ASA cannot be ruled out.

**Extended Data Fig. 6:**
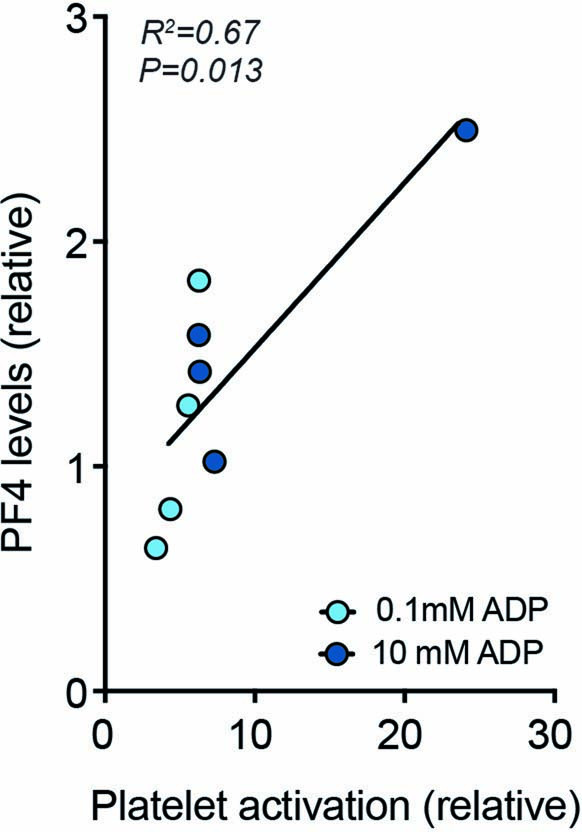
PF4 levels correlate with platelet activation (linked to Fig. 2k). Correlation between released PF4 levels and platelet activation following 0.1 mM and 10 mM ADP treatment *in vitro* onto isolated platelets. (male, age 4 months, n = 4 mice) *P = 0.013 by Linear regression. Source data

We next confirmed that platelet activation directly released factors such as PF4 (Extended Data Fig. 6), the factor most highly increased by klotho in proteomic studies (Fig. 1c). Then, we validated the proteomic finding that acute klotho treatment increased plasma PF4 using enzyme-linked immunosorbent assay (ELISA) (Fig. 2i). Following klotho treatment for over 3 weeks (Fig. 2j), PF4 was still increased by klotho (Fig. 2k). However, ASA/CPG decreased PF4 overall, and also blocked the klotho-mediated PF4 increase (Fig. 2k). It is worth noting that the rapid degradation of PF4 (with a known half-life of 2 min and 30 min; ref. ^35^), combined with the necessity for its immediate measurement, contributed to highly variable measures of the factor. It is unknown whether klotho treatment causes sustained or pulsed increases in PF4 and how this compares with other interventions such as exercise. We speculate that PF4 could act as a partial messenger of klotho-mediated cognitive enhancement.

## PF4, but not klotho, crosses into the brain

**Fig. 3:**
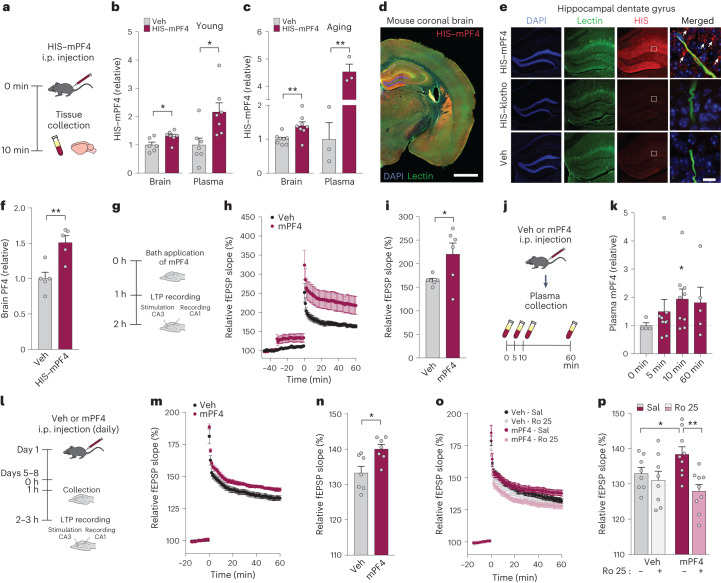
Peripherally injected PF4 is detected in the brain and enhances synaptic plasticity through NMDA receptor-dependent mechanisms. **a**, Experimental paradigm of brain tissue collection following vehicle (Veh) or HIS-tagged mPF4 injection (500 μg kg^−1^, i.p.) in young and aging mice (male, age 4 months and 18 months, *n* = 3–8 mice per group). **b**, Quantification of HIS levels in the brain and plasma of young mice, 10 min following peripheral HIS–mPF4 injection. *P = 0.030 (brain, *n* = 6 mice for Veh; *n* = 7 mice for HIS–mPF4) (two-tailed *t*-test); *P = 0.013 (plasma, *n* = 7 mice for Veh; *n* = 7 mice for HIS–mPF4) (two-tailed *t*-test). **c**, Quantification of HIS levels in the brain and plasma of old mice, 10 min following peripheral HIS–mPF4 injection. **P = 0.008 (brain, *n* = 8 mice for Veh; *n* = 8 mice for HIS–mPF4) (two-tailed *t*-test); **P = 0.001 (plasma, *n* = 3 mice for Veh; *n* = 3 mice for HIS–mPF4) (two-tailed *t*-test). **d**, Representative coronal brain tile image of young mice following peripheral HIS–mPF4 treatment. Scale bar, 1,000 μm. **e**, Representative hippocampal dentate gyrus images of young mice following Veh, HIS–klotho (500 μg kg^−1^, i.p.), or HIS–mPF4 treatment (500 μg kg^−1^, i.p.) (*n* = 3 mice as independent biological replicates per group). DAPI (blue, nuclei), lectin (green, blood vessels), HIS–klotho and HIS–mPF4 (red). White arrows indicate labeling for HIS–PF4. HIS–PF4 and HIS–klotho brightness and contrast were applied equally to their own respective controls. Scale bar, 10 μm. **f**, Quantification of mPF4 levels by PF4 ELISA in young brain following peripheral HIS–mPF4 treatment (male, age 4 months, *n* = 5 mice per group). **P = 0.005 (two-tailed *t*-test). **g**, Paradigm of hippocampal LTP recordings following mPF4 bath application. **h**, Hippocampal LTP recording of fEPSP following Veh or mPF4 bath application (male, age 3 months, *n* = 6 mice per group). **i**, Average of fEPSP over the last 10 min of recordings in young mice treated with Veh or mPF4 (*n* = 6 mice per group). *P = 0.043 (two-tailed *t*-test). **j**, Paradigm of peripheral injection of Veh or mPF4 (i.p., 20 μg kg^−1^) and plasma sample collection (male, age 4 months, *n* = 5–9 mice per group). **k**, Quantification of mPF4 levels by ELISA from plasma of young mice following its peripheral injection with either Veh (*n* = 4 mice) or mPF4 (*n* = 9 mice at 5 min, *n* = 9 mice at 10 min and *n* = 5 mice at 60 min). *P = 0.03 (two-tailed, one sample *t*-test compared with 1). **l**, Experimental paradigm of hippocampal LTP recordings from young mice treated daily with Veh or mPF4 (i.p., 20 μg kg^−1^). **m**, fEPSP recordings from acute hippocampal slices of young mice (male, age 3 months; *n* = 7 mice per group) treated with either Veh or mPF4. **n**, Average fEPSP slope over the last 10 min of recordings in young mice treated with Veh or mPF4 (*n* = 7 mice per group). *P = 0.011 (two-tailed *t*-test). **o**, Relative fEPSP recordings of acute hippocampal slices treated with Sal or Ro 25 from young mice treated with either Veh or mPF4 (male, age 3–4 months, *n* = 8 mice for Veh/Sal; *n* = 8 mice for Veh/Ro 25; *n* = 9 mice for mPF4/Sal; *n* = 9 mice for mPF4/Ro 25). **p**, Relative fEPSP slope over the last 10 min of recordings in mouse slices treated with Sal or Ro 25 from young mice peripherally injected with either Veh or mPF4 (*n* = 8 mice for Veh/Sal; *n* = 8 mice for Veh/Ro 25; *n* = 9 mice for mPF4/Sal; *n* = 9 mice for mPF4/Ro 25). Two-way ANOVA: Ro 25 *P* = 0.006; interaction PF4 by Ro 25 *P* = 0.051. *P = 0.035 (one-tailed *t*-test since replication) (Benjamini–Hochberg); **P = 0.004 (two-tailed *t*-test) (Benjamini–Hochberg). Data are presented as mean ± s.e.m. Source data

Klotho does not cross the blood–brain barrier^5,22^; since we found that klotho induces PF4 release into the blood, we tested if PF4 could reach the brain. To this end we conducted several experiments. First, we treated mice with HIS-tagged mouse PF4 (HIS–mPF4) peripherally (intraperitoneally, i.p.), collected perfused brain tissue and then measured HIS signal from the brain tissue and plasma using a HIS ELISA (Fig. 3a). In both young and aging mice, HIS–mPF4-injected mice showed increased HIS levels in the brain (1.5-fold), and highly increased levels in the plasma (2–4.5-fold), compared with vehicle-treated mice (Fig. 3a–c). These data suggest that PF4 may cross into the brain using a highly sensitive detection system.

Since we could not rule out that PF4 was sticking to blood vessels to increase levels in ELISA, we directly examined whether peripherally injected PF4 permeates the brain parenchyma using immunohistochemistry. Using whole brain imaging, we found that HIS–mPF4 was indeed detected in the brain (Fig. 3d)—with a pattern of staining similar to that of known blood–brain barrier (BBB)-permeable plasma factors^36^. In vehicle injected mice, HIS staining was absent. In mice peripherally injected with HIS–mPF4, fluorescent signal was particularly prominent in the dentate gyrus of the hippocampus, a region critical to spatial memory, and did not overlap with blood vessels (Fig. 3e). These data provide evidence that PF4 crosses the BBB and permeates the brain. Importantly, as predicted by autoradiography^22^ and immunoprecipation^5^ studies, peripherally injected HIS–klotho was not detected in the brain by immunohistochemistry (Fig. 3e), indicating specificity of the HIS–PF4 brain signal. Finally, we peripherally injected mPF4 into mice and then directly measured PF4 levels in the brain using a PF4 ELISA. In parallel with the HIS ELISA, PF4 levels increased in the brain by 1.5-fold compared with vehicle (Fig. 3f). Collectively, these data confirm that klotho does not cross the BBB and provide evidence that PF4, a putative klotho messenger, does. Since PF4 (7–8 kD) is too large for passive diffusion across the BBB, it is interesting to speculate that it undergoes adsorptive-mediated transcytosis^37,38^, a key form of BBB transport. ATM requires a cationic charge and binding to glycoproteins (which lie on the endothelial surface of the BBB), both defining properties of PF4 (refs. ^39,40^). By example, cationic proteins similar to PF4, of even larger size^41^ undergo adsorptive-mediated transcytosis. It is important to note that our data do not rule out a peripheral action of PF4 in contributing to its central nervous system functions.

## Direct PF4 application to hippocampus increases synaptic plasticity

To test if PF4 can act directly in the central nervous system and increase synaptic plasticity in the form of long term potentiation (LTP), an excitatory substrate of learning and memory^42,43^, we applied it directly to isolated hippocampal slices (Fig. 3g). We used mouse PF4 in our studies and verified that endotoxin and salinity levels of our drug solution were comparable with vehicle (phosphate-buffered saline, PBS). PF4 application to hippocampus enhanced LTP determined by field excitatory postsynaptic potential (fEPSP) recordings (Fig. 3h,i) at the CA1 Schaffer collateral pathway synapse; thus PF4 directly enhanced synaptic plasticity, even in the absence of other systemic factors it could potentially influence. These data extend findings that PF4 can have direct effects, such as increasing neurogenesis^1^, in the central nervous system. Our data suggest that peripherally injected PF4 may cross into the brain and directly enhance cognition and its substrates.

## Peripherally injected PF4 enhances synaptic plasticity through NMDAR signaling

To understand how much peripherally injected PF4 can increase its systemic levels, we measured PF4 levels by ELISA between 5 and 60 min following injection (20 μg kg^−1^, i.p.). Following this injection, plasma PF4 increased to nearly two-fold by 10 min and then began to decrease (Fig. 3j,k). This result, including the variability at each timepoint, is consistent with its estimated biphasic half-life of approximately 2 min and 30 min (ref. ^35^).

We next tested whether peripherally injected PF4, in parallel with systemic elevation of klotho^3^, and direct PF4 application to hippocampus (Fig. 3h,i), could increase LTP (Fig. 3l). We assessed LTP in acute hippocampal slices from mice that were treated daily for about 1 week with vehicle or systemic (20 μg kg^−1^, i.p.) mouse PF4 (Fig. 3l). PF4 treatment enhanced LTP determined by fEPSP recordings (Fig. 3m,n); thus, peripherally injected PF4 had central nervous system effects in enhancing synaptic plasticity.

Synaptic plasticity in this form of LTP is largely NMDAR dependent. Since klotho augments the GluN2B contribution to NMDAR signaling^3–5^ and peripherally injected PF4 recapitulated klotho-mediated synaptic enhancement in young mice, we tested whether blocking GluN2B-containing NMDARs modulates PF4 effects on synaptic plasticity. To this end, we used Ro 25-6981 (Ro 25) a GluN2B-specific antagonist with 3,000-fold specificity to GluN2B compared with other NMDAR subunits^44,45^. Acute hippocampal slices from mice were treated with either vehicle or a low dose of Ro 25 (1.5 μM). As anticipated, Ro 25 did not alter LTP in control mice treated with vehicle at the low dose (Fig. 3o,p). In contrast, low-dose Ro 25 completely abolished the PF4-induced enhancement of LTP (Fig. 3o,p). Taken together, these findings indicate that PF4, similar to klotho, engages glutamatergic signaling in young mice to enhance LTP, a substrate of learning and memory.

## PF4 enhances cognition in young and aging mice

**Fig. 4:**
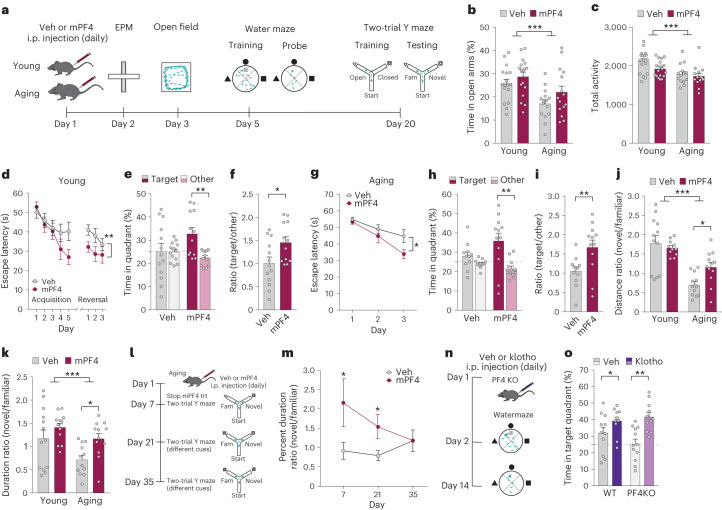
PF4 treatment enhances cognition in young mice and aging mice. **a**, Diagram of the experimental paradigm of vehicle (Veh) or mPF4 injection (i.p. 20 μg kg^−1^, daily) followed by testing in the elevated plus maze, open field testing, Morris water maze and the two-trial Y maze in young (male, age 3–5 months) and aging (male, age 17–20 months). **b**, Anxiety-like behavior was measured by percentage of time spent in the open arms of the elevated plus maze during a 10 min exploration by young (*n* = 17 mice per group) and aging (*n* = 15 mice per group) mice treated with Veh or mPF4. Two-way ANOVA: age ***P < 0.001. **c**, Total activity was measured by movements during exploration of the open field for 10 min of young (*n* = 18 mice per group) and aging (*n* = 14 mice per group) mice treated with Veh or mPF4. Two-way ANOVA: age ***P < 0.001. **d**, Spatial learning curves (platform hidden) of young mice treated with Veh (*n* = 13 mice) or mPF4 (*n* = 12 mice) in the Morris water maze. Data represent the daily average of latency to find the hidden platform over two trials. Mixed-model ANOVA for hidden training: mPF4 versus Veh, **P = 0.004 (two-tailed). **e**, Probe trial conducted 1 h after hidden platform training and removal of the escape platform (*n* = 13 mice for Veh; *n* = 12 mice for mPF4). Percentage of time the mice spent in the target quadrant of the maze, compared with the average of the other three quadrants, is shown to indicate the memory of the platform location. The dashed line represents chance performance at 25%. Two-way ANOVA: interaction *P* = 0.037; **P = 0.004 (two-tailed *t*-test) (Benjamini–Hochberg). **f**, Ratio of time young mice spent in the target quadrant relative to other quadrants of the maze (*n* = 13 mice for Veh; *n* = 12 mice for mPF4). *P = 0.024 (two-tailed *t*-test). **g**, Spatial learning curves (platform hidden) of aging mice treated with Veh or mPF4 in the water maze (*n* = 12 mice per group). Data represent the daily average of latency to find the hidden platform over four trials. Mixed-model ANOVA for hidden training: mPF4 versus Veh, *P = 0.048 (two-tailed). **h**, Probe trial conducted 1 h after hidden platform training and removal of the escape platform in aging mice (*n* = 11 mice for Veh; *n* = 12 mice for mPF4). Percentage of time mice spent in the target quadrant, compared with the average of the other three quadrants, is shown to indicate memory of the platform location. The dashed line represents chance performance at 25%. Two-way ANOVA: interaction *P* = 0.038; **P = 0.006 (two-tailed *t*-test) (Benjamini–Hochberg). **i**, Ratio of time aging mice spent in the target quadrant relative to other quadrants of the maze (*n* = 12 mice per group). **P = 0.009 (two-tailed *t*-test). **j**,**k**, Spatial and working memory of young and aging mice treated with Veh or mPF4 was assessed by the two-trial Y maze: ratio of distance traveled (*n* = 12 mice for young/Veh; *n* = 11 mice for young/mPF4; *n* = 12 mice for aging/Veh; *n* = 12 mice for aging/mPF4) (**j**) and duration in novel and familiar arms during testing was measured 16 h after training (*n* = 13 mice for young/Veh; *n* = 12 mice for young/mPF4; *n* = 12 mice for aging/Veh; *n* = 12 mice for aging/mPF4) (**k**). Two-way ANOVA: age ***P < 0.001; *P = 0.012 (two-tailed *t*-test) (Benjamini–Hochberg) (**j**), two-way ANOVA: age ***P < 0.001; *P = 0.012 (two-tailed *t*-test) (Benjamini–Hochberg) (**k**). **l**, Paradigm of Veh or mPF4 injection (i.p. 20 μg kg^−1^, daily for 7 days) in aging mice, followed by testing in the two-trail Y maze every 2 weeks. **m**, Spatial and working memory of aging mice treated with Veh or mPF4 for 7 days was assessed by the two-trial Y maze (male, age 22 months, *n* = 15 mice for Veh; *n* = 14 mice for mPF4). The ratio of the percentage of duration in the novel to the familiar arm during testing was measured 16 h after training. Two-way repeated measures ANOVA: interaction PF4 by time, *P* = 0.001; *P = 0.042 (day 7, one-tailed *t*-test since replication) (Benjamini–Hochberg); *P = 0.037 (day 21, two-tailed *t*-test) (Benjamini–Hochberg). **n**, Paradigm of Veh or klotho injection in young PF4 KO mice (males and females, age 5–7 months, *n* = 11–12 mice per group) followed by testing in water maze. **o**, Probe trial conducted 24 h after hidden platform training and removal of the escape platform (*n* = 11 mice for wild type (WT)/Veh; *n* = 11 mice for WT/klotho; *n* = 12 mice for PF4KO/Veh; *n* = 11 mice for PF4KO/klotho). The percentage time of mice spent in the target quadrant of the maze is shown to indicate their memory. Two-way ANOVA: klotho *P* < 0.001; *P = 0.050 (two-tailed *t*-test) (Benjamini–Hochberg); **P = 0.001 (two-tailed *t*-test) (Benjamini–Hochberg). Data are presented as mean ± s.e.m. Source data

**Extended Data Fig. 7:**
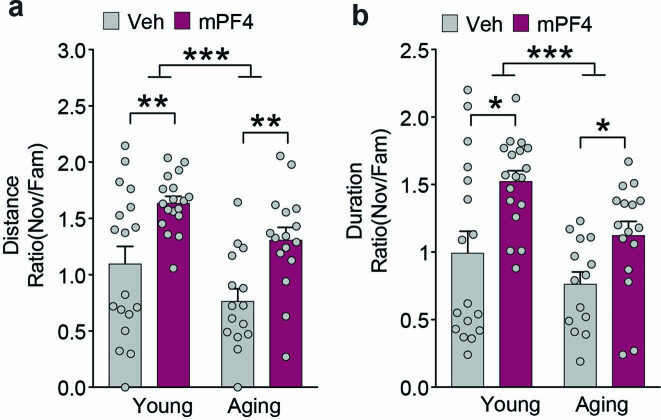
Additional mice that did not undergo watermaze were added to two-trial Y maze data shown in Fig. 4j,k. (**a**) and (**b**) With the addition of mice (watermaze naïve) to the two-trial Y maze, (final n = 14-18 mice per group), an mPF4 effect on increasing spatial and working memory in young mice became observable, in addition to its clear effect in aging mice (**a**) n = 18 mice for Young/Veh; n = 18 mice for Young/mPF4; n = 15 mice for Aging/Veh; n = 16 mice for Aging/mPF4. (**b**) n = 17 mice for Young/Veh; n = 18 mice for Young/mPF4; n = 14 mice for Aging/Veh; n = 16 mice for Aging/mPF4. Two-way ANOVA: age ***P < 0.001 (a,b); **P = 0.002 (two-tailed t-tests)(Benjamini & Hochberg)(young and aging); *P = 0.017 (two-tailed t-tests)(Benjamini & Hochberg)(young); *P = 0.020 (two-tailed t-tests)(Benjamini & Hochberg)(aging). Data are shown as means ± SEM. Source data

LTP underlies mechanisms of cognition, a highly valued manifestation of brain function. Therefore, we tested whether systemic PF4, like systemic klotho^5^, can enhance learning and memory. Young (3–5 months) and aging (17–20 months) mice were treated daily with vehicle or systemic mouse PF4 (20 μg kg^−1^, i.p.) (Fig. 4a). Aging decreased, but PF4 did not alter, anxiety-like behavior in the elevated plus maze (Fig. 4b) and total activity in the open field (Fig. 4c). In the Morris water maze, PF4 treatment improved spatial learning (Fig. 4d,g) and memory retention (Fig. 4e,f,h,i) in both young and aging mice, run with different protocols. In the two-trial Y maze, aging decreased spatial and working memory (Fig. 4j,k), a domain preferentially targeted by aging^46^. In aging, but not young, mice PF4 treatment increased exploration in the novel compared with the familiar arm, indicating that it improved deficits of cognitive aging (Fig. 4j,k). With the addition of mice (Extended Data Fig. 7a,b), the effect of PF4-mediated improvement of this cognitive domain was eventually detectable in young, but still more robust in aging. Thus, PF4 enhanced learning and memory in young and aging mice.

We performed experiments assessing for side effects of PF4 treatment including changes in weight, blood counts, blood coagulation, and liver and kidney functions—in young and aging mice following 20 days of 20 μg kg^−1^ PF4 treatment (i.p., daily) (Supplementary Table 2). We found that PF4 treatment did not alter any of these measures in young mice. In aging mice, it improved age-induced decline in kidney functions. These results indicate that the systemic PF4 treatment regimen used in this study enhanced cognition in young and aging mice without causing side effects in the outcomes measured.

To understand the duration of PF4-mediated cognitive enhancement, we tested whether short-term treatment of PF4 in aging mice induces a long-lasting effect on cognition. We injected aging mice with vehicle or PF4 daily for 1 week, stopped the treatment and then assessed cognition by the two-trial Y maze—with changed visual cues to capture new learning and memory—every 2 weeks (Fig. 4l). As expected, daily PF4 treatment of aging mice for 1 week immediately enhanced cognition (day 7) (Fig. 4m). Following this, treatment was stopped. PF4-mediated cognitive enhancement persisted for at least another 2 weeks in the absence of drug (day 21)—and was extinguished by 4 weeks (day 35) (Fig. 4m).

**Extended Data Fig. 8:**
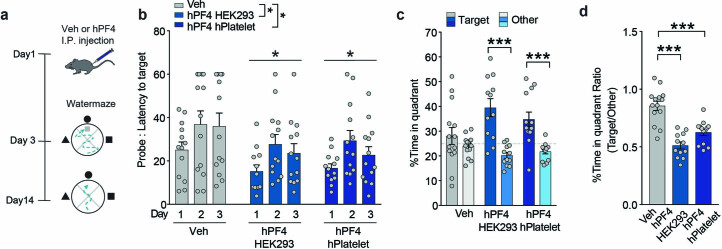
hPF4 treatment enhances cognition in mice (linked to Fig. 4). (**a**) Experimental paradigm of human PF4 treatment. Young mice (male, age 4 months, n = 12-14 mice per group) were treated with either Veh, hPF4 HEK293 (derived from a cell line), or hPF4 hPlatelet (derived from platelets) (20 μg/kg, i.p., daily) followed by testing in the watermaze. (**b**) Latency to target platform of mice (n = 12-13 mice per group) treated with Veh, hPF4 HEK23, or hPF4 hPlatelet after hidden training and removal of the escape platform. Mixed model ANOVA: *P = 0.028 (hPF4 HEK293 vs Veh)(Benjamini & Hochberg) and *P = 0.036 (hPF4 hPlatelet vs Veh)(Benjamini & Hochberg) (two-tailed) (**c**) Percentage of time the mice spent in the target quadrant 1 day after hidden training and removal of the escape platform (n = 14 mice for Veh; n = 12 mice for hPF4 HEK293; n = 13 mice for hPF4 hPlatelet). hPF4 HEK293 (Target vs Other) ***P < 0.001 (two-tailed t-test)(Benjamini & Hochberg); hPF4 hPlatelet ***P < 0.001 (two-tailed t-test) (Benjamini & Hochberg). (**d**) Ratio of time the mice spent in the target quadrant relative to other quadrants of the maze (n = 14 mice for Veh; n = 12 mice for hPF4 HEK293; n = 13 mice for hPF4 hPlatelet). One-Way ANOVA: *P* < 0.001, ***P < 0.001 (Dunnett’s test). Data are presented as means ± SEM. Source data

Our experiments utilized mouse PF4 (mPF4). Mouse and human PF4 share a 76% identity. To probe whether human PF4 (hPF4) also enhances cognition in mice, we treated mice with vehicle, hPF4 derived from a cell line, or human PF4 derived from human platelets. In parallel with mPF4, hPF4 from both sources also enhanced cognition in young mice (Extended Data Fig. 8). Since mPF4 and hPF4 enhanced cognition, but mouse protein may confer more robust species-specific biology in mice, we continued to conduct our experiments with mPF4.

Lowering PF4 levels through pharmacologic platelet inhibition (Fig. 2m) led to abrogation of klotho-mediated cognitive enhancement. However, this manipulation probably lowered several other platelet factors in tandem. Thus, we tested whether klotho specifically requires PF4 to enhance cognition. We used a colony of PF4 knockout (KO)^47^ and wild-type mice, treated them with vehicle or klotho, and tested their cognition. Klotho continued to enhance cognition (Fig. 4n,o) in PF4KO mice, regardless of sex. These results indicate that PF4 is sufficient but not necessary to recapitulate klotho-mediated cognitive enhancement. Alternatively, lifelong genetic KO of PF4 could cause compensatory increases in platelet factors with redundant biologic actions that contribute to klotho-mediated cognitive enhancement. We speculate that PF4 is one of many downstream targets of klotho and may work together with several other platelet factors to contribute to klotho-mediated cognitive enhancement.

## PF4 restores expression of aging- and cognition-associated factors in the hippocampus

**Extended Data Fig. 9:**
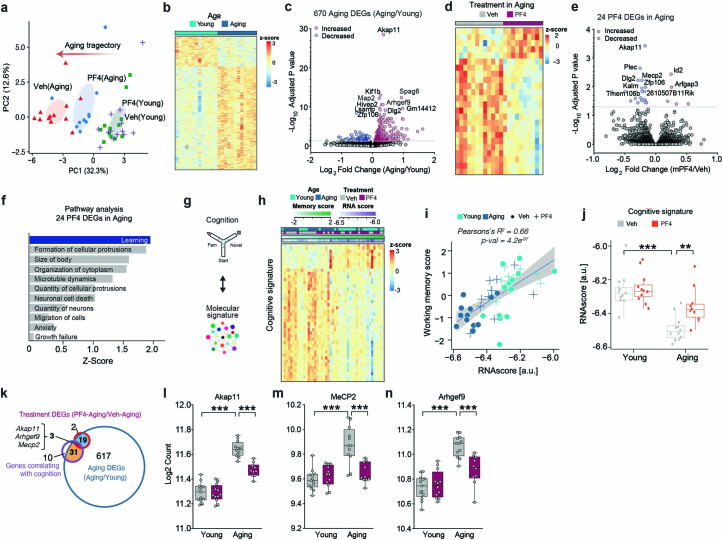
PF4 treatment restores the age-induced increase of cognition-associated factors in the aging hippocampus. **(a**) PCA analysis of experimental groups for bulk RNA sequencing shows PF4-mediated alterations in aging, but not young, hippocampal differential gene expression (DEG). Analyzed hippocampus are of young (male, 3-5 months) and aging mice (male, 17-20 months) (n = 10-13 mice per group) which were treated with Veh or mPF4 injection (i.p. 20 μg/kg, daily) and underwent behavioral experiments. (**b**) Heatmap representing age-induced alterations in the 670 DEGs. Each row represents a gene, and each column represents a biological sample. (**c**) Volcano plot showing age-induced alterations in DEGs in the hippocampus. Significant genes are labeled as increased (red) or decreased (blue). Top 10 significant genes are labeled with their respective names. Adjusted *P* value obtained by Wald test in DESeq2. (**d**) Heatmap representing PF4-induced alterations in the 24 DEGs in the aging hippocampus. Each row represents a gene, and each column represents a biological sample. (**e**) Volcano plot showing PF4-induced alterations in the aging hippocampus. Significant genes are labeled as increased (red) or decreased (blue). Top 10 significant genes are labeled with their respective names. Adjusted *P* value obtained by Wald test in DESeq2. (**f**) Functional prediction of PF4 effects on DEG of aging hippocampus by pathway analysis predicted learning. (**g**) Diagram mapping cognitive performance to hippocampal gene expression to generate a molecular signature of cognition. (**h**) Heat map of genes that comprise the molecular signature of cognition in aging hippocampus, analyzed by age, treatment, memory score, and RNA signature score. (**i**) The composite RNA score, representing the cognitive molecular signature in each mouse, correlated closely with memory scores. Correlation plot with 95% confidence interval (gray band) Two-sided Pearson’s R = 0.66, ***P < 0.001. (**j**) Aging decreased the composite RNA score represented in box and whisker plots. mPF4 treatment reversed the effect of aging on the RNA score, or the cognitive molecular signature. **P = 0.004 (Young Veh vs Aging Veh), ***P < 0.001 (Young Veh vs Aging Veh), Wilcoxon rank-sum two-sided test (Benjamini & Hochberg). (**k**) Overlap between genes significantly altered by aging, PF4, and also associated with cognition. (**l-m**) PF4 treatment restores the age-induced increase of cognition-associated genes measured by mRNA expression (RNA-seq) (male, age 3-5 months and 17-20 months, n = 10-13 mice per group). Box plots show the median and the 25–75th percentiles, and the whiskers indicate values up to 1.5-times the interquartile range. (**l**) Quantification of *AKAP11* by RNA sequencing shows aging increased expression and PF4 treatment decreased age-induced increase (n = 13 mice for Young/Veh; n = 12 mice for Young/mPF4; n = 12 mice for Aging/Veh: n = 10 mice for Aging/mPF4). Two-way ANOVA: age, treatment, and interaction *P* < 0.001; ***P < 0.001, two-tailed t-tests (Benjamini & Hochberg). (**m**) Quantification of *MeCP2* by RNA sequencing shows aging increased expression and PF4 treatment decreased age-induced increase (n = 13 mice for Young/Veh; n = 12 mice for Young/mPF4; n = 12 mice for Aging/Veh; n = 10 mice for Aging/mPF4). Two-way ANOVA: age, treatment and interaction *P* < 0.001. ***P < 0.001, two-tailed t-tests (Benjamini & Hochberg). (**n**) Quantification of *Arhgef9* by RNA sequencing shows aging increased expression and PF4 treatment decreased age-induced increase (n = 13 mice for Young/Veh; n = 12 mice for Young/mPF4; n = 12 mice for Aging/Veh; n = 10 mice for Aging/mPF4). Two-way ANOVA: treatment *P* < 0.01; age and interaction *P* = 0.001. ***P < 0.001, two-tailed t-tests (Benjamini & Hochberg). Source data

To begin identifying PF4-mediated alterations of the brain, we assessed differential gene expression in the hippocampus of young and aging mice with and without PF4 treatment. Using bulk RNA sequencing, we found by principal component analysis that PF4 treatment modified gene expression in the aging, but not young hippocampus (Extended Data Fig. 9a). Thus, we focused our analyses on aging mice. Aging significantly changed 670 genes, with most showing increased expression as shown by the heat map (Extended Data Fig. 9b,c and Supplementary Table 3). Next, we assessed if PF4 treatment alters specific gene expression in the aging hippocampus and found that it significantly alters 24 genes, with most showing decreased expression as shown by the heat map (Extended Data Fig. 9d,e and Supplementary Table 4). These gene alterations by PF4 treatment in aging robustly predicted effects on learning in pathway analysis (Extended Data Fig. 9f).

To understand the relevance of these aging- and PF4-induced gene expression changes to cognition, we mapped gene expression onto cognitive performance to generate a molecular signature of cognition (Extended Data Fig. 9g,h). We first identified a gene set containing 44 genes that are associated with cognitive function. (Extended Data Fig. 9g,h and Supplementary Table 5). The composite RNA score of this gene set is a molecular signature of cognition in individual mice. We found that this RNA score correlated closely with spatial and working memory in young and aging mice (Extended Data Fig. 9i). Aging decreased the RNA score and PF4 treatment restored it to young levels (Extended Data Fig. 9j). Thus, PF4 treatment reversed the effect of aging on a cognitive signature of hippocampal gene expression.

We then examined the convergence and overlap of PF4- and aging-induced gene alterations with cognition-associated genes. Their intersection revealed three factors (Extended Data Fig. 9k): *Akap11*, *Arhgef9* and *Mecp2* (Extended Data Fig. 9l,m,n). Aging increased expression of these factors in the hippocampus—and PF4 treatment restored the age-induced increase close to young levels (Extended Data Fig. 9l,m,n). It is worth noting that each of these target candidates of PF4 in aging is abundant in the brain^48^ and relevant to human brain disorders linked with cognition. *AKAP11* variants associate with neuropsychiatric disease^49^; *ARHGEF9* mutations cause intellectual disability^50^; and *MECP2* mutations also cause severe intellectual disability through loss of function in Rett’s syndrome^51^ and increased dosage in MECP2 duplication syndrome^51,52^, with MECP2 overexpression impairing cognition and synaptic transmisssion^52^. Whether the increased expression of these individual factors in aging is compensatory, causal to age-induced deficits, or neither remains to be determined—as does PF4’s role in modulating their expression.

Our study has several limitations and caveats. In interpreting the link between klotho and platelets, the overall levels of platelet activation were low and klotho agonism was mild compared with agonists involved in thrombosis and wound healing. Despite this caveat, klotho reproducibly activated platelets and we speculate that low levels of agonism and activation might release chemokines without blood clotting. This remains to be further investigated. In understanding the relationship between PF4 and klotho, the persistent klotho-mediated cognitive enhancement in the absence of PF4 may not support a major and required role for this protein in klotho action. While PF4 recapitulated klotho-mediated cognitive enhancement, it was not necessary. We speculate that klotho may have several downstream targets, including other platelet factors with similar biologic activity; however, the evidence to support this is indirect. Despite this caveat, the finding that ASA/CPG acutely decreases PF4—probably in tandem with other platelet factors—and blocks klotho-mediated cognitive enhancement strongly suggests that platelets are required. Several strengths of our study include an untargeted approach to identifying platelet factors as possible targets of klotho, new evidence of PF4 brain permeability, and the findings of PF4-mediated synaptic and cognitive enhancement in several cohorts. Our findings open myriad questions including how klotho activates platelets, which factors klotho requires for cognitive enhancement, what mechanisms PF4 engages to permeate the brain, what forms (monomeric, oligomeric and fibrillar) are necessary for PF4-mediated cognitive enhancement, what receptors PF4 binds in the brain and whether PF4 requires the select factors identified for cognitive enhancement in aging. It will also be important in future studies to decipher the mechanisms of protective versus deleterious platelet activation states.

In summary, our study reveals an unconventional role for platelets, through PF4, in enhancing cognition in the young and aging brain. Klotho activated platelets in a manner similar to exercise^1^. Since exercise increases klotho^53^, it is interesting to speculate that their respective signaling pathways for platelet activation, yet to be determined, could converge. Our findings suggest that platelets can act as circulating messengers that modulate cognition itself through release of factors such as PF4. Our data suggest that platelet activation was required, and PF4—which crossed into the brain—was sufficient, to recapitulate klotho mediated-cognitive enhancement. PF4 enhanced cognition in young mice and engaged synaptic mechanisms of glutamatergic signaling. In aging mice, PF4 also improved cognition and restored age-induced increases in select factors associated with cognition. Augmenting platelet factors may enhance cognition in the young brain and counteract cognitive deficits in the aging brain.

## Methods

### Mice

All mice were on a congenic C57BL/6J background and kept on a 12 h light/dark cycle, humidity of 30–70% and temperature 68–79° F with ad libitum access to food and water. The standard housing group was five mice per cage except for single housing during Morris water maze studies. Sex and ages of mice used are indicated in legends. Cognitive and behavioral studies were carried out during the light cycle. All platelet activation assays, ELISAs, cognitive and behavioral studies, and synaptic plasticity experiments were conducted blinded to genotype and treatment during study execution and analysis, unless indicated otherwise. All other data collection and analysis were not performed blind to the conditions. Homozygous PF4 KO was previously generated and characterized as described^47^. In brief, the coding region for mouse PF4 was replaced with a 1.8 kb neomycin resistance gene. No randomization method was used to allocate animals to experimental groups. Allocation of mice or samples in the organization of experimental conditions considered genotype, drug treatment, sex if indicated, age and distribution within cage. All studies were approved by the Institutional Animal Care and Use Committee of the University of California, San Francisco, and conducted in compliance with National Institutes of Health (NIH) guidelines.

### Drug treatment

Mouse α-Klotho (klotho) (R&D, 1819-KL) was diluted in PBS (pH 7.5) and injected subcutaneously (s.c.) at a volume of 10 µl g^−1^ (adjusted to weight of mouse) at a dose of 10 µg kg^−1^ 4 h before samples were collected for plasma proteomic analysis, mPF4 ELISA and platelet activation assays. mPF4 (PROSPEC, CHM-245) and hPF4 (PROSEPC, CHM-234 and Genscript, Z03026) were diluted in PBS (pH 7.5) and administered i.p. at a volume of 20 µl g^−1^ (adjusted to weight of mouse) 1 h before each day of training and testing at a dose of 20 µg kg^−1^. Recombinant proteins were used within 1 week of thawing from −80 °C stock solutions and stored at 4 °C.

### Plasma proteomic profiling

Plasma samples were prepared and analysed by data-independent acquisition liquid chromatography–mass spectrometry at Biognosys using a 2 h segmented gradient as previously described^54^. For spectral library generation, 12 high-pH reversed phase chromatography fractions were analyzed by data-dependent acquisition liquid chromatography–mass spectrometry and searched against a mouse protein database (Uniprot without isoforms, 2018-07-01) using SpectroMine software (Biognosys). Data-independent acquisition data were analyzed with Spectronaut Pulsar X software (Biognosys), and data were filtered for a detection false discovery rate <1% on peptide and protein level. Peptide intensities were normalized using local regression normalization as implemented in Spectronaut. On average 505 protein groups (14,341 peptide precursors) were quantified in each run, and 535 protein groups (18,447 peptide precursors) were quantified at least once across the samples. Statistical analysis between vehicle- and klotho-treated samples was performed in Spectronaut using default settings and controlled for using Benjamini–Hochberg correction.

### Platelet activation and count by flow cytometry

Platelet activation states were measured using flow cytometry as described^1^ with minor modifications. Briefly, whole blood via cardiac puncture was collected into a final concentration of 0.38% sodium citrate solution (pH 7) and then centrifuged at 200*g* for 10 min at room temperature. Equal volume of plasma from each mouse was collected and transferred to a new tube with Hanks’ Balanced Salt Solution (HBSS) (with ethylenediaminetetraacetic acid, pH 6.4) and then centrifuged at 1,200*g* for 20 min at room temperature. The platelet pellet was resuspended in HBSS (pH 6.4) and then stained with CD61–PE (1:6, Thermo Fisher) and CD62P–Alexa 647 (1:6, BD Bioscience) antibodies for 30 min at room temperature. Platelets stained with platelet marker and activation marker were resuspended in the FACS buffer (PBS with 1% bovine serum albumin and 1% sodium azide (pH 6.4)) to give enough dilution so that very small size platelet can be detected when flowing through FACS machine. A total of 10,000 events were analyzed by flow cytometry at a flow rate of 25 µl ml^−1^. Activated platelets were identified as CD62P-positive cells within the CD61-positive population. Platelet count, assessed by platelet concentration, was calculated as the total number of CD61-positive platelets per each sample. Platelet count was normalized to the volume read by FACS. For in vitro studies, platelets isolated from mice were resuspended in HBSS (pH 6.4) and then were treated with vehicle, klotho or 1 mM ADP for 2 min at 37 °C. The immunostained platelets with CD61–PE and CD62P–Alexa 647 were analyzed by flow cytometry.

### Platelet inhibition treatment

ASA (Bayer Health Care) and CPG (Sciegen) diluted in drinking water (ASA 0.4 mg ml^−1^, CPG 0.15 mg ml^−1^) were supplied ad libitum from 72 h before the start of testing to the end of testing. Control mice received regular drinking water^31,55^.

### ELISA

For measurement of mPF4, ELISAs (R&D Systems) were performed according to the manufacturer’s directions. Briefly, each plasma sample was diluted by with ELISA buffer and analyzed for mPF4 by ELISA. For measurement of HIS-tagged mouse PF4 (GeneTex, GTX00334-pro), the HIS-tagged protein was first diluted in PBS (pH 7.5) and administered at a dose of 500 μg kg^−1^ (i.p.) 10 min before perfusion and brain tissue collection. Hippocampal and cortical tissues were dissected and then lysed with RIPA buffer to obtain homogenate samples. To determine HIS signal in the homogenates, HIS ELISA (Cell Biolabs) was performed according to the manufacturer’s directions. Briefly, 10 μg of protein from the brain homogenate sample was diluted with ELISA buffer and analyzed for HIS by ELISA.

### Immunohistochemistry

Mice were perfused with cold PBS (10 ml min^−1^) for 4 min using peristaltic pump. Whole brains were isolated and post-fixed in 4% (w/v) paraformaldehyde for 48 h before preservation in 30% (w/v) sucrose in PBS. Whole brains were sectioned coronally at a thickness of 40 μm on a freezing sliding microtome. Sections were stored in the cryoprotective medium at −20 °C. Free-floating sections were blocked with donkey serum and incubated with primary antibodies at 4 °C overnight at the following concentration for microscopy: rabbit anti-HIS (1:200, Invitrogen MA5-33032) and fluorescein-labeled lectin (1:200, Vector Laboratories). After washing, sections were incubated with donkey anti-rabbit Alexa Fluor Plus 555 (1:200, Thermo Fisher, A32794) and 300 nM of 4′,6-diamidino-2-phenylindole (DAPI) at room temperature for 2 h (refs. ^36,56^). Sections were washed, and mounted with Vectashield before imaging on digital fluorescent microscope with spinning disk confocal microscope (Nikon CSU-W1) For brain tile imaging, sections were imaged on the widefield microscope (Nikon 6D).

### Electrophysiology

Coronal brain slices of 300 μm thickness from mice were obtained as described^3–5^, with minor modifications. Briefly, measurements were obtained from the CA1 region following stimulation of the Schaffer Collateral path. Mice were anesthetized with isoflurane and the brain was collected and immediately placed in ice-cold artificial cerebrospinal fluid containing the following (in millimolar): 124 NaCl, 2.8 KCl, 2 MgSO_4_, 1.25 NaH_2_PO_4_, 10 glucose, 26 NaHCO_3_, 2.5 CaCl_2_, 1.3 ascorbic acid and sliced on a vibratome (Leica). Slices were incubated at 32 °C for 30 min, then recovered at room temperature for 1 h before testing. Slices were transferred to an interface chamber with circulating, oxygenated (95% O_2_ and 5% CO_2_) artificial cerebrospinal fluid at 30 °C and left to recover for 10–15 min before any stimulation. For field potential recordings, acute hippocampal slices were placed on a Med64-Quad II multielectrode array (Alpha MED Scientific). fEPSPs were elicited and recorded via planar electrodes of the Quad II 2×8 Probe AL-MED-PG501A by aligning the electrodes and the stratum radiatum region of hippocampal slices. An input–output curve was performed at the beginning of each recording to determine the appropriate stimulation intensity. Test stimuli at 30% of maximal intensity were delivered at 0.05 Hz and a stable baseline of fEPSP of 15–20 min was established before LTP induction. LTP was induced using a theta-burst protocol comprised of two trains delivered every 20 seconds, each train containing 10 bursts at 5 Hz, each burst containing four pulses at 100 Hz. Ro 25 (Tocris) was dissolved in normal saline at 2 mg ml^−1^ and added to the perfusion of acute hippocampal slices 1 h before the fEPSP recordings. Recordings and analysis were performed using Med64 Mobius Software (Alpha MED Scientific).

### Serum/urine chemistries and hematology

Serum and urine chemistry profiles were obtained using the AU680 Chemistry System (Beckman Coulter) with reagents designed for the AU680 Chemistry System, and end-product concentrations (either enzymatic end-point or enzymatic kinetic) were determined spectrophotometrically. Vitamin D analysis was performed at Cornell Veterinary Diagnostics Lab using a 25-hydroxyvitamin D radioimmunoassay from serum samples. Serum FGF23 level was determined by FGF23 ELISA (Immutopics, cat. no. 60-6800) according to the manufacturer’s directions. Complete blood count was obtained from ethylenediaminetetraacetic-acid-anticoagulated whole blood samples by use of the flow cytometry-based Sysmex XT-2000iV Automated Hematology Analyzer (Sysmex). White blood cell count, red blood cell count and platelet count were obtained for each sample. When there was a discrepancy between the manual differential cell counts and automated differential cell counts, the former was reported and used for further data analysis. Prothromin time and activated partial thromboplastin clotting time measurements were obtained using the Stago STA automated coagulation from sodium citrate plasma samples.

### Behavioral and cognitive tasks

#### Y maze

Mice were tested in the Y maze as described^3–5,57^. Briefly, mice were placed in one arm of the Y maze with three identical arms, 120° apart and allowed to explore freely for 5 min. Arm entries were recorded (AnyMaze) and an alternation was counted any time the mouse entered each of the three arms in successive arm entries. Percent alternations were measured.

#### Elevated plus maze

Mice were tested in the elevated plus maze as described^3–5,57^. After habituation to dim lighting in the testing room, mice were placed at the center of the apparatus (Hamilton-Kinder) at the junction between open and closed arms of the maze and allowed to explore for 10 min. Time spent in open arms, an index of anxiety-like behavior, was measured.

#### Open field test

Mice were tested in the open field as described^3–5,57^. Total activity of mice in the open field was measured with an automated Flex-Field/Open Field Photobeam Activity System (San Diego Instruments). Mice were tested in a clear plastic chamber for 10 min, with two photobeam arrays measuring movements.

#### Morris water maze

Mice were tested in the Morris water maze (Noldus Ethovision) as described^3–5,57^. Briefly, the water maze pool (diameter, 122 cm) contained white, opaque water (21 ± 1 °C). A square, 14 cm^2^ platform was submerged 2 cm below the water surface. One day before the hidden platform training, mice underwent two trials of pre-training by swimming through a channel to mount a hidden platform. During hidden platform training, the platform was placed in the same location and the mice drop location was varied between trials. Mice received two or four training trials per day, daily for 3–5 days, as indicated. Mice were allowed to search for the platform for a maximum of 60 s per trial. Following hidden platform training, the probe trial was conducted during the platform was removed. Mice were allowed to search for the platform location, an indication of memory, for 60 s.

#### Two-trial Y maze

Mice were tested in the two-trial Y maze (Noldus Ethovision), which assesses spatial and working memory, as described^5^. Briefly, mice underwent training by exploring the maze with a visual cue in one arm and another arm blocked off. Sixteen hours after training, mice underwent testing with all three arms open (start arm, familiar arm and novel arm) and the distance traveled exploring the novel arm compared with the familiar arm, an index of memory, was measured.

### Bulk RNA sequencing

RNA was extracted and the complementary DNA library was prepared from snap-frozen hippocampus as described^58^. Briefly, Illumina sequencing libraries were prepared using NextSeq Hi Output KT v2.5 and sequenced on an iSeq 100 sequencing system. Samples with fewer than 1 million reads were excluded because of low coverage. Data visualization and analysis was done using custom R scripts and the ‘deseq2’,’ggplot2’, ‘dlpyr’, ‘VISION’ and ‘pheatmap’ Bioconductor packages were used. Briefly, raw counts were normalized using Deseq2 and principal component analysis revealed an age and treatment effect. Differentially expressed genes were identified using the standard Deseq2 pipeline. *P* values were adjusted for multiple testing and genes with padj <0.05 were called statistically significant. A cognitive *z*-score for each mouse was used as a covariate and the mice were binned into ‘High’ or ‘Low’ categories using their cognitive *z*-scores. The transcriptional cognitive signature was determined by identifying a set of genes differentially regulated in ‘High’-performing mice compared to ‘Low’-performing mice. An RNA score based on the signature was calculated for each mouse. Hierarchical clustering was performed using Ward’s clustering algorithm to generate an expression heat map based on their cognitive *z*-scores. Ingenuity pathway analysis was used for identifying Gene Ontology terms that most associate with the differentially expressed genes in old mice treated with mPF4 (QIAGEN).

### Statistics and reproducibility

Statistical analyses were executed with GraphPad Prism (version 7.0) or R for *t*-tests and analyses of variance (ANOVAs). Data distribution was tested for normality using the Shapiro–Wilk test; variance of data was not formally tested. Differences between two means were assessed by two-tailed *t*-tests for all experiments unless indicated otherwise. One-tailed *t*-tests were applied in an unbiased manner to experiments that were independent replications of previous findings because of prior knowledge of the expected direction of change. Differences among multiple means were assessed by two-way ANOVA. R was used for Wilcoxon rank-sum tests. Unless indicated otherwise, multiple comparisons of post hoc *t*-tests were corrected with the Benjamini–Hochberg. A mixed-model ANOVA was used for analyses of Morris water maze data and included the effects of repeated measures. Linear mixed-effects models were fit in R^59^ using the standard lme4 (ref. ^60^) package. Statistics are summarized in Supplementary Table 6. In mouse studies, exclusion criteria (greater than two standard deviations above or below the mean) were defined a priori to ensure unbiased exclusion of outliers. No statistical methods were used to pre-determine sample sizes but our sample sizes are similar to those reported for behavior and synaptic plasticity in our previous publications^3–6,21^. Error bars represent ± standard error of the mean (s.e.m.). Null hypotheses were rejected at or below a *P* value of 0.05.

### Reporting summary

Further information on research design is available in the Nature Portfolio Reporting Summary linked to this article.

### Supplementary information

Reporting Summary

Supplementary Tables 1–6 Supplementary Tables 1–6.

### Source data

Source Data Fig. 1 Statistical source data.

Source Data Fig. 2 Statistical source data.

Source Data Fig. 3 Statistical source data.

Source Data Fig. 4 Statistical source data.

Source Data Extended Data Fig. 2 Statistical source data.

Source Data Extended Data Fig. 3 Statistical source data.

Source Data Extended Data Fig. 4 Statistical source data.

Source Data Extended Data Fig. 5 Statistical source data.

Source Data Extended Data Fig. 6 Statistical source data.

Source Data Extended Data Fig. 7 Statistical source data.

Source Data Extended Data Fig. 8 Statistical source data.

Source Data Extended Data Fig. 9 Statistical source data.

## Data Availability

Plasma proteomics raw data are available from ProteomeXchange Consortium via the PRIDE with dataset identifier PXD040167. RNA-sequencing raw data are available from the Gene Expression Omnibus under accession code GSE171929. All other data supporting the findings of this study are available in the source data files or from the corresponding author upon reasonable request.

## Code availability

All the code for RNA-sequencing analysis is available at https://github.com/DenaDubal/Park_et_al_2023.

## References

[1] Leiter O (2019). Exercise-induced activated platelets increase adult hippocampal precursor proliferation and promote neuronal differentiation. Stem Cell Rep..

[2] van der Meijden PEJ, Heemskerk JWM (2019). Platelet biology and functions: new concepts and clinical perspectives. Nat. Rev. Cardiol..

[3] Dubal DB (2014). Life extension factor klotho enhances cognition. Cell Rep..

[4] Dubal DB (2015). Life extension factor klotho prevents mortality and enhances cognition in hAPP transgenic mice. J. Neurosci..

[5] Leon J (2017). Peripheral elevation of a klotho fragment enhances brain function and resilience in young, aging, and α-synuclein transgenic mice. Cell Rep..

[6] Castner, S. A. et al. Longevity factor klotho enhances cognition in aged nonhuman primates. *Nat. Aging*10.1038/s43587-023-00441-x (2023). 10.1038/s43587-023-00441-x PMC10432271 37400721

[7] Masso A, Sanchez A, Bosch A, Gimenez-Llort L, Chillon M (2018). Secreted αKlotho isoform protects against age-dependent memory deficits. Mol. Psychiatry.

[8] Leiter O, Walker TL (2019). Platelets: the missing link between the blood and brain?. Prog. Neurobiol..

[9] Chateau MT, Araiz C, Descamps S, Galas S (2010). Klotho interferes with a novel FGF-signalling pathway and insulin/Igf-like signalling to improve longevity and stress resistance in *Caenorhabditis elegans*. Aging.

[10] Kurosu H (2005). Suppression of aging in mice by the hormone klotho. Science.

[11] Singh AP (2019). αKlotho regulates age-associated vascular calcification and lifespan in zebrafish. Cell Rep..

[12] Laszczyk AM (2017). Klotho regulates postnatal neurogenesis and protects against age-related spatial memory loss. Neurobiol. Aging.

[13] Zhao Y (2020). Klotho overexpression improves amyloid-β clearance and cognition in the APP/PS1 mouse model of Alzheimer’s disease. Aging Cell.

[14] Wolf I (2008). Klotho: a tumor suppressor and a modulator of the IGF-1 and FGF pathways in human breast cancer. Oncogene.

[15] Urakawa I (2006). Klotho converts canonical FGF receptor into a specific receptor for FGF23. Nature.

[16] Liu H (2007). Augmented Wnt signaling in a mammalian model of accelerated aging. Science.

[17] Yokoyama JS (2017). Systemic klotho is associated with KLOTHO variation and predicts intrinsic cortical connectivity in healthy human aging. Brain Imaging Behav..

[18] Gaitan JM (2022). Circulating klotho is higher in cerebrospinal fluid than serum and elevated among KLOTHO heterozygotes in a cohort with risk for Alzheimer’s disease. J. Alzheimers Dis..

[19] Yokoyama JS (2015). Variation in longevity gene KLOTHO is associated with greater cortical volumes. Ann. Clin. Transl. Neurol..

[20] Kundu P (2022). Serum levels of α-klotho are correlated with cerebrospinal fluid levels and predict measures of cognitive function. J. Alzheimers Dis..

[21] Gupta S (2022). KL1 domain of longevity factor klotho mimics the metabolome of cognitive stimulation and enhances cognition in young and aging mice. J. Neurosci..

[22] Hu MC (2016). Renal production, uptake, and handling of circulating αklotho. J. Am. Soc. Nephrol..

[23] Maugeri, N. et al. Platelet microparticles sustain autophagy-associated activation of neutrophils in systemic sclerosis. *Sci. Transl. Med.*10.1126/scitranslmed.aao3089 (2018). 10.1126/scitranslmed.aao3089 30045975

[24] Baumann J (2022). Reduced platelet forces underlie impaired hemostasis in mouse models of MYH9-related disease. Sci. Adv..

[25] Lefrancais E (2017). The lung is a site of platelet biogenesis and a reservoir for haematopoietic progenitors. Nature.

[26] Jiang L (2013). A critical role of thrombin/PAR-1 in ADP-induced platelet secretion and the second wave of aggregation. J. Thromb. Haemost..

[27] Schoenwaelder SM (2016). 14-3-3ζ regulates the mitochondrial respiratory reserve linked to platelet phosphatidylserine exposure and procoagulant function. Nat. Commun..

[28] Iglesias MJ (2023). Elevated plasma complement factor H related 5 protein is associated with venous thromboembolism. Nat. Commun..

[29] Pircher J (2018). Cathelicidins prime platelets to mediate arterial thrombosis and tissue inflammation. Nat. Commun..

[30] Xiao, Z. et al. FGF23 expression is stimulated in transgenic α-Klotho longevity mouse model. *JCI Insight*10.1172/jci.insight.132820 (2019). 10.1172/jci.insight.132820 PMC6962016 31801907

[31] Lieschke F, Zheng Y, Schaefer JH, van Leyen K, Foerch C (2020). Measurement of platelet function in an experimental stroke model with aspirin and clopidogrel treatment. Front. Neurol..

[32] Zheng Y (2019). Dual antiplatelet therapy increases hemorrhagic transformation following thrombolytic treatment in experimental stroke. Stroke.

[33] Sangkuhl K, Klein TE, Altman RB (2010). Clopidogrel pathway. Pharmacogenet. Genomics.

[34] Awtry EH, Loscalzo J (2000). Aspirin. Circulation.

[35] Dubrac A (2010). Functional divergence between 2 chemokines is conferred by single amino acid change. Blood.

[36] Yang AC (2020). Physiological blood–brain transport is impaired with age by a shift in transcytosis. Nature.

[37] Terstappen GC, Meyer AH, Bell RD, Zhang W (2021). Strategies for delivering therapeutics across the blood–brain barrier. Nat. Rev. Drug Discov..

[38] Herve F, Ghinea N, Scherrmann JM (2008). CNS delivery via adsorptive transcytosis. AAPS J..

[39] Visentin GP, Moghaddam M, Beery SE, McFarland JG, Aster RH (2001). Heparin is not required for detection of antibodies associated with heparin-induced thrombocytopenia/thrombosis. J. Lab. Clin. Med..

[40] Rauova L (2006). Role of platelet surface PF4 antigenic complexes in heparin-induced thrombocytopenia pathogenesis: diagnostic and therapeutic implications. Blood.

[41] Rhea EM (2021). The S1 protein of SARS-CoV-2 crosses the blood–brain barrier in mice. Nat. Neurosci..

[42] Morris RG, Anderson E, Lynch GS, Baudry M (1986). Selective impairment of learning and blockade of long-term potentiation by an *N*-methyl-d-aspartate receptor antagonist, AP5. Nature.

[43] Nakazawa K, McHugh TJ, Wilson MA, Tonegawa S (2004). NMDA receptors, place cells and hippocampal spatial memory. Nat. Rev. Neurosci..

[44] Lu, W., Du, J., Goehring, A. & Gouaux, E. Cryo-EM structures of the triheteromeric NMDA receptor and its allosteric modulation. *Science*10.1126/science.aal3729 (2017). 10.1126/science.aal3729 PMC5568803 28232581

[45] Paoletti P, Neyton J (2007). NMDA receptor subunits: function and pharmacology. Curr. Opin. Pharmacol..

[46] Bizon JL, Foster TC, Alexander GE, Glisky EL (2012). Characterizing cognitive aging of working memory and executive function in animal models. Front. Aging Neurosci..

[47] Eslin DE (2004). Transgenic mice studies demonstrate a role for platelet factor 4 in thrombosis: dissociation between anticoagulant and antithrombotic effect of heparin. Blood.

[48] Consortium GT (2020). The GTEx Consortium atlas of genetic regulatory effects across human tissues. Science.

[49] Palmer DS (2022). Exome sequencing in bipolar disorder identifies AKAP11 as a risk gene shared with schizophrenia. Nat. Genet..

[50] Hines DJ (2022). Human ARHGEF9 intellectual disability syndrome is phenocopied by a mutation that disrupts collybistin binding to the GABAA receptor α2 subunit. Mol. Psychiatry.

[51] Chahrour M (2008). MeCP2, a key contributor to neurological disease, activates and represses transcription. Science.

[52] Na ES (2012). A mouse model for MeCP2 duplication syndrome: MeCP2 overexpression impairs learning and memory and synaptic transmission. J. Neurosci..

[53] Amaro-Gahete FJ (2018). Role of exercise on S-klotho protein regulation: a systematic review. Curr. Aging Sci..

[54] Bruderer R (2017). Optimization of experimental parameters in data-independent mass spectrometry significantly increases depth and reproducibility of results. Mol. Cell Proteomics.

[55] Lauer A (2011). Antiplatelet pretreatment does not increase hematoma volume in experimental intracerebral hemorrhage. J. Cereb. Blood Flow Metab..

[56] De Miguel Z (2021). Exercise plasma boosts memory and dampens brain inflammation via clusterin. Nature.

[57] Davis, E. J. et al. A second X chromosome contributes to resilience in a mouse model of Alzheimer’s disease. *Sci. Transl. Med*. 10.1126/scitranslmed.aaz5677 (2020). 10.1126/scitranslmed.aaz5677 PMC8409261 32848093

[58] Schaum N (2020). Ageing hallmarks exhibit organ-specific temporal signatures. Nature.

[59] R: a language and environment for statistical computing. Version 3.6.1 (R Foundation for Statistical Computing, 2019).

[60] Bates D, Mächler M, Bolker B, Walker S (2015). Fitting linear mixed-effects models using lme4. J. Statist. Softw..

