# Supplementary material for: Platelet factors are induced by longevity factor klotho and enhance cognition in young and aging mice
Source: Nat Aging. 2023 Aug 16;3(9):1067–78. doi: 10.1038/s43587-023-00468-0 (PMC10501899; doi:10.1038/s43587-023-00468-0)
Supplement: Supplementary file 1 — Reporting Summary [file 43587_2023_468_MOESM1_ESM.pdf]

nature portfolio

Corresponding author(s): Dena Dubal

Last updated by author(s): 7/7/2023

Reporting Summary

Nature Portfolio wishes to improve the reproducibility of the work that we publish. This form provides structure for consistency and transparency in reporting. For further information on Nature Portfolio policies, see our Editorial Policies and the Editorial Policy Checklist.

Statistics

For all statistical analyses, confirm that the following items are present in the figure legend, table legend, main text, or Methods section.

n/a	Confirmed	
☐	☒	The exact sample size (*n*) for each experimental group/condition, given as a discrete number and unit of measurement
☐	☒	A statement on whether measurements were taken from distinct samples or whether the same sample was measured repeatedly
☐	☒	The statistical test(s) used AND whether they are one- or two-sided<br>*Only common tests should be described solely by name; describe more complex techniques in the Methods section.*
☒	☐	A description of all covariates tested
☐	☒	A description of any assumptions or corrections, such as tests of normality and adjustment for multiple comparisons
☐	☒	A full description of the statistical parameters including central tendency (e.g. means) or other basic estimates (e.g. regression coefficient) AND variation (e.g. standard deviation) or associated estimates of uncertainty (e.g. confidence intervals)
☐	☒	For null hypothesis testing, the test statistic (e.g. *F*, *t*, *r*) with confidence intervals, effect sizes, degrees of freedom and *P* value noted<br>*Give P values as exact values whenever suitable.*
☒	☐	For Bayesian analysis, information on the choice of priors and Markov chain Monte Carlo settings
☒	☐	For hierarchical and complex designs, identification of the appropriate level for tests and full reporting of outcomes
☐	☒	Estimates of effect sizes (e.g. Cohen's *d*, Pearson's *r*), indicating how they were calculated

Our web collection on statistics for biologists contains articles on many of the points above.

Software and code

Policy information about availability of computer code

Data collection	AnyMaze(version 6.35), Ethovision (version 10, Noldus), Attune Nxt Acoustic Focusing Cytometer, Med64 Mobius (Alpha MED Scientific Inc), SpectroMine (version 2.0), AU680 Chemistry System, Sysmex XT-2000iV Automated Hematology Analyzer, Stago STA, NextSeq Hi Output KT v2.5, iSeq 100
Data analysis	Proteomic analyses were carried out with Spectronaut Pulsar X (version 15). Image analyses were carried out with ImageJ1.53t. Statistical analyses were carried out with GraphPad Prism (version 7.0) or R (version 4.2.1).

For manuscripts utilizing custom algorithms or software that are central to the research but not yet described in published literature, software must be made available to editors and reviewers. We strongly encourage code deposition in a community repository (e.g. GitHub). See the Nature Portfolio guidelines for submitting code & software for further information.

Data

Policy information about availability of data

All manuscripts must include a data availability statement. This statement should provide the following information, where applicable:

- Accession codes, unique identifiers, or web links for publicly available datasets
- A description of any restrictions on data availability
- For clinical datasets or third party data, please ensure that the statement adheres to our policy

Plasma proteomics raw data are available from ProteomeXchange Consortium via the PRIDE with dataset identifier PXD040167.

RNA-seq raw data are available from the Gene Expression Omnibus under accession code GSE171929.
All code for RNA seq analysis is available at https://github.com/DenaDubal/Park_et_al_2023

Research involving human participants, their data, or biological material

Policy information about studies with human participants or human data. See also policy information about sex, gender (identity/presentation), and sexual orientation and race, ethnicity and racism.

Reporting on sex and gender	N/A
Reporting on race, ethnicity, or other socially relevant groupings	N/A
Population characteristics	N/A
Recruitment	N/A
Ethics oversight	N/A

Note that full information on the approval of the study protocol must also be provided in the manuscript.

Field-specific reporting

Please select the one below that is the best fit for your research. If you are not sure, read the appropriate sections before making your selection.

☒ Life sciences ☐ Behavioural & social sciences ☐ Ecological, evolutionary & environmental sciences

For a reference copy of the document with all sections, see nature.com/documents/nr-reporting-summary-flat.pdf

Life sciences study design

All studies must disclose on these points even when the disclosure is negative.

Sample size	No statistical methods were used to pre-determine sample sizes but our sample sizes are similar to those reported for behavior and synaptic plasticity in our previous publications.
Data exclusions	In the murine biochemistry, synaptic plasticity, and behavioral studies, exclusion criteria (greater than 2 SDs above or below the mean) were defined a priori to ensure unbiased exclusion of outliers. Examples of some outliers in studies included a clotted blood sample, mice that floated instead of swam, synaptic plasticity that returned to baseline and did not maintain amplitude.
Replication	Significant results are replicated in ELISA, platelet activation assay, immunohistochemistry, synaptic plasticity and behavior studies. Replication includes either at least two independent cohorts or tests.<br>Plasma proteomics and hippocampal RNA seq analysis was not attempted for replication. However, PF4 elevation results from plasma proteomics were confirmed by ELISA. Synaptic plasticity studies with direct mPF4 application to hippocampus was not attempted for replication.
Randomization	No randomization method was used to allocate animals to experimental groups
Blinding	We blind experimenters to genotype and/or drug treatment for studies.

Reporting for specific materials, systems and methods

We require information from authors about some types of materials, experimental systems and methods used in many studies. Here, indicate whether each material, system or method listed is relevant to your study. If you are not sure if a list item applies to your research, read the appropriate section before selecting a response.

Materials & experimental systems

n/a	Involved in the study
☐	☒ Antibodies
☒	☐ Eukaryotic cell lines
☒	☐ Palaeontology and archaeology
☐	☒ Animals and other organisms
☒	☐ Clinical data
☒	☐ Dual use research of concern
☒	☐ Plants

Methods

n/a	Involved in the study
☒	☐ ChIP-seq
☐	☒ Flow cytometry
☒	☐ MRI-based neuroimaging

Antibodies

Antibodies used: CD61-PE (Thermo Fischer, 12-0611-82), CD62P-alexa 647 (BD Bioscience, 563674) , rabbit anti-HIS (Invitrogen MA5-33032), fluorescein-labelled lectin (Vector Laboratories), Donkey anti-rabbit Alexa Fluor Plus 555 (Thermo Fischer, A32794)

Validation: CD61-PE (Thermo Fischer, 12-0611-82), CD62-alexa 647 (BD Bioscience, 563674) have been validated by Thermo Fischer and BD Bioscience, respectively by demonstrating Facs analysis on mouse platelets responsive to thrombin treatment. These antibodies were also validated in other study (Leiter et al, 2019, Stem Cell Rep), where they were shown to specifically label activated mouse platelets through FACS analysis. Anti-Lectin was validated for the indicated species and applications by the manufacturer and in another study (Yang et al, 2020, Nat). Anti-HIS was shown to specifically stain his-tagged protein in the immunostaining with no cross reactivity with other endogenous protein in mammalian or bacteria cells by the manufacturer.

Animals and other research organisms

Policy information about studies involving animals; ARRIVE guidelines recommended for reporting animal research, and Sex and Gender in Research

Laboratory animals: C57BL/6J mice studies were conducted in males. PF4KO studies were conducted in female and male mice. Young mice were 3-6 months old. Aging mice were 17-22 months old.

Wild animals: The study did not involve wild animals

Reporting on sex: C57BL/6J mice studies were conducted in males. PF4KO studies were conducted in female and male mice

Field-collected samples: The study did not involve samples collected from the field

Ethics oversight: All studies were approved by the Institutional Animal Care and Use Committee of the University of California, San Francisco, and conducted in compliance with NIH guidelines.

Note that full information on the approval of the study protocol must also be provided in the manuscript.

Flow Cytometry

Plots

Confirm that:

☒ The axis labels state the marker and fluorochrome used (e.g. CD4-FITC).

☒ The axis scales are clearly visible. Include numbers along axes only for bottom left plot of group (a 'group' is an analysis of identical markers).

☒ All plots are contour plots with outliers or pseudocolor plots.

☒ A numerical value for number of cells or percentage (with statistics) is provided.

Methodology

Sample preparation: Briefly, whole blood via cardiac puncture was collected into a final concentration of 0.38% sodium citrate solution (pH 7) and then centrifuged at 200g for 10min at room temperature. Equal volume of plasma from each mouse was collected and transferred to a new tube with HBSS (with EDTA, pH 6.4) and then centrifuged at 1200g for 20min at room temperature. The platelet pellet was resuspended in HBSS (pH 6.4) and then stained with CD61-PE (Thermo Fischer) and CD62-alexa 647 (BD Bioscience) antibodies for 30min at room temperature. Platelets stained with platelet marker and activation marker were resuspended in the FACS buffer (PBS with 1% BSA and 1% sodium azide (pH 6.4)) to give enough dilution so that very small size platelet can be detected when flowing through FACS machine.

Instrument: Attune NxT

Software	Attune Nxt Acoustic Focusing Cytometer was used to collect and analyzed the data.
Cell population abundance	cell sorting not employed.
Gating strategy	Using the SSC/CD61 gating, debris was removed by gating on the main cell population. CD61-positivity and CD62P-negativity thresholds for the resting platelet population was defined on the basis of unstained samples. Identical positivity and negativity thresholds was applied to all samples.

☒ Tick this box to confirm that a figure exemplifying the gating strategy is provided in the Supplementary Information.
